# Weight perception and its association with socio-demographic and health-related factors among Korean adolescents

**DOI:** 10.1186/s12889-015-2624-2

**Published:** 2015-12-24

**Authors:** Anna Shin, Chung Mo Nam

**Affiliations:** Department of Public Health, College of Medicine, Yonsei University, Seoul, South Korea; Department of Preventive Medicine/Department of Biostatistics, College of Medicine, Yonsei University, 50 Yonsei-ro, Seodaemun-Gu, Seoul, 120-752 South Korea

**Keywords:** Weight perception, Overestimation, Underestimation, Misperception, Korean adolescents

## Abstract

**Background:**

Adolescence is a time of rapid growth with dramatic changes in physical appearance. The body image established at this time could affect their physical and mental health throughout their entire life. However, adolescents sometimes perceive themselves as underweight or overweight irrespective of actual weight status. The purpose of the present study was to examine the extent of weight misperception for Korean adolescents, to explore socio-demographic factors associated with weight misperception, and to examine gender-specific differences in the relationships between weight misperception and health-related factors.

**Methods:**

We selected data on 3321 adolescents aged 12–18 years from the five-year Korea Health and Nutrition Examination Survey (KNHANES) datasets (2007–2011). Self-perceived weight status was compared with measured weight status by cross-tabulation. The generalized logit model was used to explore the socio-demographic factors associated with weight misperception, and separate logistic regression models were fitted to examine gender-specific differences in the relationships between weight misperception and health-related factors.

**Results:**

Overall, 25.8 % of boys (overestimation 17.1 %; underestimation 8.6 %) and 29.3 % of girls (overestimation 24.0 %; underestimation 5.3 %) misclassified their weight status according to the objective standards. Weight overestimation was particularly prominent among underweight girls. Weight misperception was associated with socio-demographic factors such as gender, age, BMI, place of residence, and maternal education level. Weight overestimation and underestimation in boys and weight overestimation in girls were significantly related to inappropriate weight control practice. However, weight underestimation in girls seems to be negatively linked to inappropriate weight control practice.

**Conclusions:**

Based on the results of the present study, comprehensive intervention programs for adolescents and their parents could be devised to raise self-awareness of their weight status, to overcome weight misperception, and to prevent obesity and its related health risks.

## Background

Adolescence is a time of rapid growth with dramatic changes in physical appearance [1]. The body image established at this time could affect their physical and mental health throughout their entire life [1]. A previous study indicates that adolescents are often split between those who desire to lose weight versus those who desire to gain weight [2]. This means that adolescents sometimes perceive themselves as underweight or overweight irrespective of actual weight status. Weight misperception is the discordance between an individual’s actual weight status and the perception of his/her weight [3]. According to a recent study, body size misperception was a strong predictor of body dissatisfaction regardless of actual weight status [4].

Weight misperception among adolescents is affected by socio-demographic and environmental factors including gender, body mass index (BMI), ethnicity, socioeconomic status (SES), and media exposure [5–10]. It is also associated with health-related factors, such as weight control practice and psychosocial impairment [11–13]. Previous studies suggested that body weight/shape concerns were potential mediators of the association between obesity and psychosocial impairment [14, 15]. These findings indicate the need to target weight perception in the clinical management of obesity in order to improve both physical health and psychosocial outcomes [14]. Thus, weight misperception is a meaningful variable in obesity prevention and treatment for adolescents.

Weight misperception is a prevalent concern among adolescents in both Western and Eastern countries [5, 16, 17]. According to the 1999–2007 Youth Risk Behavior Survey data for US adolescents, 29-33 % of overweight adolescents misperceived their weight [18]. A Korean study using the 2011 Korea Youth Risk Behavior Web-based Survey-VII data revealed that the prevalence of weight misperception was 49.3 % (overestimation, 23.7 %; underestimation, 25.6 %) [6].

Meanwhile, significant gender differences in weight perception and weight control practices have been reported. Girls tend to overestimate their weight, while boys tend to underestimate their weight [6, 7, 11, 19, 20]. A thin body (e.g., low waist-hip ratio, low volume-height index) is associated with female bodily attractiveness [21], whereas a v-shaped upper body (e.g., low waist-chest ratio), which is related to muscularity, is associated with male bodily attractiveness [21]. Thus, boys and girls may be striving for a gender-specific ideal of bodily attractiveness, and perceive themselves to be falling short of the ideal.

Much of the existing research has been confined to studies focusing on weight misperception and its association with socio-demographic factors among adolescents recruited from Western populations. Previous Korean studies mostly investigated weight misperception patterns using BMI calculated from self-reported height and weight. Besides, limited studies have been performed to explore health-related factors associated with weight misperception based on gender. Therefore, the purpose of the present study was to quantify the extent of weight misperception for a nationally representative sample of South Korean adolescents using BMI based on anthropometric measurements, to explore socio-demographic factors associated with weight misperception, and to examine gender-specific differences in the relationships between weight misperception and health-related factors. We hypothesized that socio-demographic factors including gender, age, BMI, household income, residence, maternal obesity and maternal education level, might influence weight misperception among adolescents, and that adolescents who misperceive their weight might be more likely to have inappropriate health-related outcomes than those who perceive their weight accurately.

## Methods

### Study population and database

This study used data obtained from the Korea National Health and Nutrition Examination Survey (KNHANES) conducted from 2007 through 2011, which was a cross-sectional and nationally representative survey examining the health and nutrition status of South Koreans. The target population of KNHANES comprises non-institutionalized Korean citizens residing in Korea. The sampling plan follows a multi-stage clustered probability design [22]. The surveys included the Health Interview Survey, Health Examination Survey, and Nutrition Survey. The health interview and health examination are performed by trained medical staff and interviewers at the mobile examination center, and dieticians’ visits to the homes of the study participants are followed up. According to standardized protocols, all health examination procedures are performed by trained medical personnel and all equipment is calibrated periodically [22]. More details regarding the survey design and methods have been provided elsewhere [23].

From the five-year KNHANES datasets (2007–2011), we selected data regarding 3868 adolescents aged 12–18 years, including their demographic characteristics, household environment, socioeconomic status, health status, and parents’ health status. However, 547 participants were excluded because of lack of information about their parents, family composition, body mass index, and weight perception. Consequently, we included 3321 adolescents aged 12–18 years for the present study. Ethical approval was obtained from the Institutional Review Board of Yonsei University’s Graduate School of Public Health. We received a waiver of informed consent because the data were obtained from a public database.

### Measurements

#### Classification of objective weight status

Objective weight status was determined according to a respondent’s body mass index (BMI; weight in kilograms divided by the square of the height in meters), which was based on anthropometric measurements from the Health Examination Survey. The cut-points for weight classification by clinical standards are as follows: underweight (BMI < 5^th^ percentile), normal (5^th^ ≤ BMI < 85^th^ percentile), overweight (85^th^ ≤ BMI < 95^th^ percentile), and obese (BMI ≥ 95^th^ percentile for age and gender, using the 2007 Korean Pediatric Growth Charts) [24].

#### Classification of body weight perception

For a measure of self-perceived weight status, respondents’ perceptions about their own weight status were assessed through the question: “How do you describe your weight?” Answers were chosen from “very underweight,” “a little underweight,” “about the right weight,” “a little overweight,” and “very overweight.”

To compare objective weight status with self-perceived weight status, the categories of “a little underweight” and “about the right weight” were combined into a “normal weight” category. ‘Very underweight’ and ‘very overweight’ of subjective standards correspond to ‘underweight’ and ‘obese’ of objective standards, respectively. Participants were then placed into one of three categories: correct perception (self-perception is concordant with the objective standard), underestimate (self-perception is in a lighter category than the objective standard), and overestimate (self-perception is in a heavier category than the objective standard).

#### Socio-demographic factors

The annual household income, which was based on reports from household heads during the Health Interview Survey, was chosen as a socio-demographic factor. The household income levels were divided into 4 quartiles based on the national median household income: lowest (<25^th^ percentile), middle−low (25–49^th^ percentile), middle−high (50–74^th^ percentile), and highest (≥75^th^ percentile for median household income). Place of residence was classified, according to the national administration system, as living in an urban area or not (e.g., living in a town or in the countryside). Maternal obesity was defined as a BMI at or above 25 for a child’s mother. The survey results of maternal educational levels were classified into 3 categories: 1) middle school; 2) high school; 3) college completion.

#### Health-related factors

Weight control practice during the past year was assessed with participants allowed to give four possible responses: I tried to 1) lose weight; 2) stay the same weight; 3) gain weight; or 4) I did nothing about my weight. For useful interpretation, the answer was reclassified into three categories: 1) appropriate (e.g., trying to gain weight in underweight individuals, trying to maintain weight in normal weight individuals, trying to lose weight in overweight/obese individuals), 2) inappropriate (e.g., trying to maintain or lose weight in underweight individuals, trying to lose or gain weight in normal weight individuals, trying to maintain or gain weight in overweight/obese individuals), or 3) do nothing. “Moderate intensity exercise” was defined as those lasting at least 10 min and that increased heart rate slightly. Examples included slow swimming, doubles tennis, volleyball, or occupational or recreational activity involving the carrying of light objects (i.e., having greater intensity than walking). This definition was based on the health interview questionnaire of KNHANES. Participants were asked how many days during the last seven days you had participated in moderate exercises. Answers were grouped into three categories: ‘never’, ‘1−2 days/week’, or ‘≥3 days/week’. Regarding stress, the following question was asked: “How much stress do you feel on daily basis?” Answers were chosen from ‘very little’, ‘little’, ‘much’, and ‘very much’. Smoking and drinking experience were assessed by asking: “Have you ever tried cigarette smoking/drinking alcohol, even once?” Participants were grouped into yes/no categories.

### Statistical analysis

Data in all statistical analyses for our study were weighted to account for the complex sampling design of the KNHANES. Self-perceived weight status was compared with objective weight status based on BMI by cross-tabulation. To measure agreement between objective weight status and self-perceived weight status, kappa statistics based on the survey weighted cell percentages were calculated.

To explore gender-specific differences in socio-demographic factors associated with weight misperception, the generalized logit model (PROC SURVEYLOGISTIC in SAS) was used, after accounting for primary sample units, stratification, and sample weights from the KNHANES. We first selected socio-demographic variables including age, gender, BMI, household income, place of residence, number of family members, family generation type, maternal/paternal age, maternal/paternal education level, maternal/paternal occupation, and maternal/paternal obesity from the 5-year dataset. To determine relevant variables for a regression model, we then used chi-square tests (PROC SURVEYFREQ in SAS) for comparing differences among three weight perception groups (correct, underestimate, overestimate groups) in the chosen categorical variables. As a result, the factors showing a statistically significant difference (*p* < 0.05) were chosen for a multinomial logistic regression, provided that age, BMI, household income, and maternal educational level were included irrespective of its *p*–value. Correct weight perception served as reference for comparison.

To examine gender-specific differences in the relationships between weight misperception and health-related factors, we fitted separate logistic regression models (PROC SURVEYLOGISTIC in SAS). We first selected health-related variables including weight control practice, moderate intensity exercise, severe intensity exercise, walking exercise, suicide ideation, depression experience, feeling under stress, sleeping hours, drinking experience, and smoking experience from the 5-year dataset. To determine relevant variables for logistic regression models, we then used chi-square tests for comparing differences among three weight perception groups (correct, underestimate, overestimate groups) in the chosen variables. Based on these analyses, five variables that exhibited a statistically significant relationship (*p* < 0.05) were chosen for separate logistic regression models (weight control practice, moderate intensity exercise, feeling under stress, drinking experience, and smoking experience as outcome variables). Age, BMI, household income, and place of residence were controlled for in each model. Because ten independent predictions were tested in the gender-stratified analysis, the Bonferroni correction was used to reduce the chances of obtaining false-positive results (type 1 errors); *P* − values < 0.005 were considered statistically significant. Statistical analyses were performed using the software SAS version 9.2 (SAS Institute, Cary, NC, USA).

## Results

### Agreement between objective weight status and self-perceived weight status

Table 1 presents the general characteristics of the study participants according to sex. Boys had a higher BMI level than girls (*p* < 0.001). There were significant differences according to sex in health-related factors such as weight control practice, moderate exercise, feeling under stress, smoking and drinking experience (*p* < 0.001). Table 2 displays a cross-tabulation of objective weight status with self-perceived weight status for boys and girls. In the normal weight group (5^th^ − 85^th^ percentile), 76.5 % of boys and 76.2 % of girls believed that they were approximately the right weight, or considered themselves to be a little underweight. In the underweight group (<5^th^ percentile), 57.9 % of boys believed that they were very underweight, whereas 26.7 % of girls considered themselves to be very underweight. Moreover, 56.2 % of obese boys (≥95^th^ percentile) and 54.9 % of obese girls considered themselves to be very overweight.

**Table 1:** Sample characteristics^a,b^

Variable	Boys	Girls	All
	(n = 1754)	(n = 1567)	(n = 3321)
Age (years)	15.02 (0.05)	15.03 (0.06)	15.02 (0.04)
Body mass index (BMI)	21.27 (0.10)	20.67 (0.11)	20.99 (0.08)
Household income quartile			
Lowest	11.01	12.64	11.76
Middle-low	25.23	27.35	26.21
Middle-high	32.00	30.67	31.38
Highest	31.76	29.34	30.64
Residence			
Urban area	83.16	82.54	82.87
Rural area	16.84	17.46	17.13
Maternal weight status			
Underweight	3.62	3.68	3.65
Normal weight	71.36	70.62	71.02
Obese	25.02	25.70	25.34
Maternal educational level			
≤ Middle school	16.71	16.44	16.59
High school	57.42	56.44	56.96
≥ College	25.87	27.13	26.45
Weight control practice			
Lose weight	25.71	47.65	35.90
Stay the same weight	13.16	16.05	14.50
Gain weight	18.08	2.95	11.05
Do nothing	43.04	33.35	38.54
Moderate exercise			
Never	43.70	61.18	51.83
1-2 days/week	28.40	25.21	26.92
≥3 days/week	27.90	13.60	21.26
Feeling under stress			
Very little	16.22	13.03	14.74
Little	59.09	54.23	56.83
Much	21.28	27.56	24.20
Very much	3.40	5.18	4.23
Smoking experience			
Yes	23.52	10.51	17.48
No	76.48	89.49	82.52
Drinking experience			
Yes	41.24	34.73	38.22
No	58.76	65.27	61.78

^a^For categorical variables, cell percentage (%) was a weighted percentage using survey sample weights

^b^For continuous variables, mean and standard error of means were provided

**Table 2:** Comparison of objective weight status with self-perceived weight status^a,b,c,d^

Self-perceived status	Objective status (Percentile of BMI)				
	(P < 5^th^)	(5^th^ ≤ P < 85^th^)	(85^th^ ≤ P < 95^th^)	(P ≥ 95^th^)	Total^e^
	% (SE)	% (SE)	% (SE)	% (SE)	% (SE)
Boys					
Very underweight	**4.48 (0.60)**	4.93 (0.62)	—	—	9.41 (0.82)
A little underweight/ about right	3.26 (0.47)	**56.89 (1.41)**	1.05 (0.25)	0.08 (0.06)	61.29 (1.35)
A little overweight	—	12.35 (0.99)	**9.48 (0.82)**	2.55 (0.41)	24.39 (1.18)
Very overweight	—	0.20 (0.14)	1.33 (0.30)	**3.38 (0.53)**	4.91 (0.60)
Total^e^	7.74 (0.75)	74.37 (1.29)	11.86 (0.89)	6.02 (0.66)	
Girls					
Very underweight	**1.96 (0.38)**	1.00 (0.29)	—	—	2.96 (0.48)
A little underweight/ about right	5.27 (0.66)	**56.86 (1.51)**	1.08 (0.27)	—	63.21 (1.45)
A little overweight	0.10 (0.10)	15.61 (1.07)	**8.02 (0.80)**	3.19 (0.52)	26.92 (1.33)
Very overweight	—	1.20 (0.33)	1.83 (0.38)	**3.88 (0.58)**	6.91 (0.74)
Total^e^	7.33 (0.78)	74.66 (1.27)	10.94 (0.93)	7.07 (0.86)	

^a^SE, standard error

^b^P, Percentile of BMI for age and gender, using the 2007 Korean Pediatric Growth Charts

^c^The cell percentages (%) are weighted percentages using survey sample weights

^d^Bold numbers indicate correct perception

^e^The total percentage of objective or subjective measured weight status

Overall, 25.8 % of boys and 29.3 % of girls misclassified their weight status, according to the objective standards. Among these misclassification cases, 17.1 % of boys and 24.0 % of girls overestimated their weight, while 8.6 % of boys and 5.3 % of girls underestimated their weight. Based on survey-weighted cell percentages, the kappa statistic (*k*) is 0.49 for boys and 0.40 for girls, suggesting a moderate agreement between objective and self-perceived weight status.

### Factors associated with weight status misperception

Weight misperception was associated with socio-demographic factors such as gender, age, BMI, place of residence and maternal educational level, as shown in Table 3. Regarding gender, girls were less likely to underestimate their body weight compared to boys (OR = 0.70, *p* = 0.040), and were more likely to overestimate their body weight (OR = 1.47, *p* = 0.001). Age also had significant effects. In older girls, the probability of underestimating their weight gets lower (OR = 0.77, *p* = 0.002) and the probability of overestimating gets higher (OR = 1.14, *p* = 0.001). However, in older boys, the probability of overestimating gets lower (OR = 0.91, *p* = 0.017). The BMI was associated with weight misperception: the higher the BMI, the greater the chance of boys underestimating and overestimating their weight (underestimate OR = 1.10, *p* = 0.004; overestimate OR = 1.07, *p* = 0.001) and the greater the chance of girls underestimating their weight (OR = 1.36, *p* < 0.001).

**Table 3:** Factors associated with weight status misperception from multinomial logistic regression^a,b^

Variables	Boys and girls		Boys		Girls	
	Underestimate	Overestimate	Underestimate	Overestimate	Underestimate	Overestimate
	OR (95 % CI)	OR (95 % CI)	OR (95 % CI)	OR (95 % CI)	OR (95 % CI)	OR (95 % CI)
Gender						
Boys (ref)						
Girls	0.70 (0.49–0.98)*	1.47 (1.17–1.85)**				
Age	0.93 (0.85–1.01)	1.02 (0.96–1.08)	0.98 (0.88–1.09)	0.91 (0.83–0.98)*	0.77 (0.65–0.90)**	1.14 (1.06–1.23)**
BMI	1.18 (1.12–1.24)***	1.05 (1.02–1.08)**	1.10 (1.03–1.17)**	1.07 (1.03–1.11)**	1.36 (1.23–1.50)***	1.02 (0.98–1.07)
Household income quartile						
Lowest (ref)						
Middle-low	1.04 (0.54–2.01)	0.82 (0.53–1.26)	1.06 (0.40–2.80)	0.61 (0.33–1.13)	0.89 (0.39–2.01)	1.06 (0.59–1.89)
Middle-high	0.77 (0.40–1.48)	0.83 (0.55–1.27)	0.93 (0.36–2.38)	0.74 (0.40–1.37)	0.47 (0.19–1.12)	0.97 (0.55–1.70)
Highest	0.85 (0.44–1.63)	0.83 (0.54–1.29)	0.94 (0.37–2.39)	0.79 (0.41–1.50)	0.59 (0.23–1.52)	0.95 (0.53–1.68)
Place of residence						
Urban area (ref)						
Rural area	1.25 (0.80–1.94)	0.76 (0.54–1.06)	1.02 (0.52–2.00)	1.08 (0.66–1.77)	1.48 (.78–2.81)	0.55 (0.35–0.86)**
Maternal obesity						
No (ref)						
Yes	1.16 (0.83–1.62)	1.09 (0.86–1.38)	1.16 (0.74–1.82)	1.22 (0.85–1.76)	1.05 (0.59–1.87)	1.03 (0.74–1.43)
Maternal educational level						
≤ Middle school (ref)						
High school	1.68 (1.00–2.82)	1.15 (0.85–1.57)	1.52 (0.80–2.88)	1.17 (0.73–1.88)	2.29 (1.05–5.01)*	1.19 (0.77–1.83)
≥ College	1.55 (0.87–2.77)	1.03 (0.71–1.49)	1.72 (0.86–3.42)	1.01 (0.58–1.77)	1.43 (.55–3.73)	1.06 (0.66–1.71)

*p < 0.05; **p < 0.01; ***p < 0.001

^a^Odds ratio refers to the odds of incorrect perception of weight status (overestimate or underestimate)

^b^Gender was removed in the gender-stratified analysis

Place of residence showed significant effects on misperception as well. Girls living in a rural area were less likely to overestimate their weight (OR = 0.55, *p* = 0.009). Regarding maternal educational level, girls whose mother graduated from high school were more likely to underestimate their body weight (OR = 2.29, *p* = 0.037).

### Associations between weight misperception and health-related factors

Table 4 shows that weight misperception was associated with weight control practice among both boys and girls. Compared with boys who perceived their weight accurately, boys who underestimated or overestimated their weight were more likely to attempt to gain or lose weight inappropriately (underestimate OR = 2.51, *p* < 0.001; overestimate OR = 1.90, *p* = 0.003). In girls, weight overestimation was positively related to inappropriate weight control practice (OR = 2.69, *p* < 0.001). However, girls who underestimated their weight were less likely to attempt to lose or gain weight inappropriately, although the result was not statistically significant according to the Bonferroni correction (OR = 0.42, *p* = 0.035).

**Table 4:** Associations (OR and 95 % CI) between weight misperception and health-related factors among Korean adolescents^a,b,c^

Outcome variables	Boys		Girls	
	Underestimate	Overestimate	Underestimate	Overestimate
	OR (95 % CI)	OR (95 % CI)	OR (95 % CI)	OR (95 % CI)
Weight control practice				
Appropriate (ref)				
Inappropriate	2.51 (1.47–4.30)**	1.90 (1.25–2.90)**	0.42 (0.19–0.94)*	2.69 (1.81–3.98)**
Do nothing	1.02 (0.60–1.72)	0.90 (0.59–1.38)	1.01 (0.49–2.08)	1.70 (1.11–2.63)*
Moderate exercise				
Never (ref)				
1-2 days/week	0.79 (0.49–1.27)	1.02 (0.72–1.44)	1.02 (0.53–1.96)	0.98 (0.69–1.39)
≥3 days/week	0.81 (0.48–1.36)	1.11 (0.76–1.63)	0.68 (0.28–1.65)	0.92 (0.61–1.40)
Feeling under stress				
Very little (ref)				
Little	0.75 (0.24–2.35)	1.43 (0.55–3.72)	2.08 (0.50–8.71)	1.26 (0.61–2.57)
Much	1.49 (0.76–2.92)	1.55 (0.89–2.68)	1.55 (0.57–4.27)	1.39 (0.85–2.27)
Very much	1.13 (0.64–2.01)	1.26 (0.79–2.01)	1.63 (0.65–4.10)	1.17 (0.74–1.84)
Smoking experience				
No (ref)				
Yes	1.47 (0.92–2.36)	1.05 (0.70–1.58)	3.03 (1.21–7.58)*	1.15 (0.70–1.89)
Drinking experience				
No (ref)				
Yes	1.09 (0.72–1.66)	0.74 (0.51–1.06)	1.01 (0.55–1.84)	1.37 (0.99–1.89)

*p < 0.05; **p < 0.005

^a^Separate logistic regression models were fit for each outcome variable Correct weight perception (accurate) as reference

^b^Age, BMI, household income, and place of residence were controlled for in the models

^c^Because of multiple comparisons (*n* = 10), a *p*-value < 0.005 was considered significant using Bonferroni correction

## Discussion

### Agreement between objective weight status and self-perceived weight status

Our study revealed that girls had greater misperception of their weight status compared with boys. Unlike previous studies that boys tend to underestimate their weight, while girls tend to overestimate their weight [7, 11, 19, 20], both boys and girls in the present study were more likely to overestimate than underestimate their weight. Our results might be attributed to the fact that prolonged exposure to slim ideal images in mass media had a profound effect on the desire to be thinner among adolescent boys as well as girls. The results also showed that weight overestimation was particularly prominent among underweight girls. Inconsistent with our findings, weight misperception in Western countries was mainly observed in overweight adolescents, possibly because a larger proportion of adolescents are overweight in Western than in Eastern nations [20]. Thus, the difference in the distribution of weight misperception might be due to the differences in ethnic composition and overweight prevalence rate of the sample.

In previous Korean studies, to measure agreement between objective weight status and self-perceived weight status, ‘overweight’ and ‘obesity’ grouped together for the purpose of the analysis rather than considering obesity as a separate category [6, 11]. However, we used four standardized clinical categories (underweight, normal, overweight, and obesity) as their objective classification schemes. For this reason, the misclassification of weight seems to be lower in our study compared to the previous Korean studies.

### Factors associated with weight status misperception

According to our findings, girls were more likely than boys to overestimate and less likely to underestimate their body weight. Particularly among older girls, the probability of underestimating their body weight gets lower and the probability of overestimating gets higher. These trends are consistent with many previous studies [7, 11, 19, 20]. Adolescence is an important period for body image and self-concept development. Prolonged pressure from their social and cultural environment may affect adolescents’ weight norms, and may play an important role in adolescents setting their own weight standards, no matter whether these standards are healthy or unrealistic [25, 26]. Thus, adolescents seem to become eager for a sex-specific ideal of bodily attractiveness as they age [1]. Therefore, educational efforts that focus on healthy body weight and accurate weight perception may be particularly beneficial for adolescent girls.

BMI was another factor linked to weight misperception. In South Korea, being tall is something to envy regardless of gender, and growth-promoting attempts by parents are widespread [27]. Usually, an obsession with being tall was more serious in boys. In our study, more than two thirds of the misclassification cases in boys and more than half of the misclassification cases in girls were in normal weight groups. If normal weight boys with higher BMI consider tall and slim body to be healthy, then they could overestimate their weight. If, on the other hand, they consider tall and muscular body to be desirable, then they could underestimate their weight. Therefore, without an individualized approach for the misclassification cases in each weight group, any interventional plan is more likely doomed to failure.

Place of residence was also associated with weight misperception. Only girls living in a rural area appeared less likely to overestimate their weight. In rural areas of Korea, the proportion of elderly people is relatively large [28]. According to a previous study, in Asian third-generation families, grandparents may also exert an influence on adolescent health behaviors [29]. Likewise, comments and attitudes towards health by elderly people living in the community may affect adolescents’ weight norms. Maternal educational level and household income were analyzed as indices of the participants’ socioeconomic status (SES), and as a result maternal educational level was a significant variable among girls. A previous study found that Chinese girls who had highly educated parents were less likely to misperceive themselves as being overweight [7]. However, a study using US representative data has shown no significant relationship between weight misperception and SES [20]. Therefore, further studies are needed to establish evidence for an association between weight perception and SES.

### Associations between weight misperception and health-related factors

Our study revealed that girls’ overestimation of their weight was related to inappropriate weight control practice. Girls’ underestimation seems to be negatively linked to inappropriate weight control practice. In our culture, thin models are hailed as the ideal body type, and these images become the standards by which the individual defines herself and others. These standards of thinness and beauty may make many adolescent girls feel overweight [30], and finally they may attempt to lose weight inappropriately. However, girls who underestimate their weight might feel relatively relieved and satisfied with their body shape, and in the end, they could be less likely to practice for weight control inappropriately.

On the other hand, both weight underestimation and overestimation were associated with inappropriate weight control practice among boys. Boys are divided between those who desire to lose weight and those who wish to gain weight and musculature. Muscularity may be idealized in boys and so, being underweight may be as likely to be stigmatized as being overweight [31]. Even normal weight boys could attempt to gain weight inappropriately because of a desire for a muscular body. Meanwhile, for adolescents grappling with emerging pubertal changes, comparing themselves to the stars of stage and screen is unavoidable [1]. For this reason, even normal weight boys could perceive themselves as being overweight, and could attempt to lose weight for a lean, muscular body inappropriately.

In line with our results, previous studies revealed that weight overestimation or underestimation was related to unhealthy weight control practices [18, 32]. Based on an understanding of gender-based differences in weight misperception patterns and its associated factors among adolescents, comprehensive health promotion programs, particularly school-based or family-based obesity prevention programs, including regular body weight screening, checking on weight perception status, appropriate physical activities and health education could be devised for adolescents and their parents. Especially, from a clinical perspective, health professionals need to be aware of the effects of weight misperception on physical and psychological health when counseling underweight or overweight adolescents.

Moderate exercise, stress, smoking and drinking experience were not associated with weight misperception in our study. However, according to previous studies, inaccurate weight perception in adolescents was negatively associated with physical activity [33, 34]. In addition, severe overestimation or underestimation was positively related to substance use such as smoking and drinking experience [34]. Therefore, further studies are needed to understand the relationship between weight misperception and health-related factors.

### Study strengths and limitations

First, this study was based on recent nationally representative data. Second, because our study examined gender-specific differences in the relationship between weight misperception and its associated factors, effective interventional strategies based on gender could be devised. Third, our study used measured weight and height to accurately classify BMI indices for this representative sample of adolescents.

However, there are several limitations. First, the definition of the variables used in our study was based on the health interview questionnaire of the KNHANES, but weight control practice was defined operationally for useful interpretation and meaningful analysis. Second, this study is comparing two different scales (the scale of objective weight status and that of self-perceived weight status) and presenting them as if they are comparable. Self-perception is more concerned with body image and body attractiveness, whereas objective categories are more concerned with health. Therefore, caution is needed in interpretation of our findings. Lastly, we cannot explain the cause-and-effect relationship between these variables because of the study’s cross-sectional nature.

## Conclusion

Both boys and girls were more likely to overestimate than underestimate their weight. Weight overestimation was particularly prominent among underweight girls. Weight misperception was associated with socio-demographic factors such as gender, age, BMI, place of residence, and maternal education level, and there was a gender-specific difference in the association between weight misperception and inappropriate weight control practice. Based on an understanding of weight misperception patterns among adolescents and their association with socio-demographic and health-related factors, comprehensive intervention programs for adolescents and their parents could be devised to raise self-awareness of their weight status, to overcome weight misperception, and to prevent obesity and its related health risks.
